# Complete Genome Sequence of a Bioactive Pseudomonas sp. Strain, DTU12.3, Isolated from Soil in Denmark

**DOI:** 10.1128/MRA.00121-19

**Published:** 2019-04-18

**Authors:** Pavelas Sazinas, May Iren Aune, Marie Højmark Fischer, Jonas Greve Lauritsen, Lone Gram, Lars Jelsbak

**Affiliations:** a Department of Biotechnology and Biomedicine, Technical University of Denmark, Kongens Lyngby, Denmark; University of Maryland School of Medicine

## Abstract

Here, we report the complete annotated genome sequence of a Pseudomonas sp. strain, DTU12.3. It was isolated from leaf-covered soil in Denmark and potentially has bioactivity against certain plant pathogens.

## ANNOUNCEMENT

The Pseudomonas genus consists of a large number of species that are able to inhabit a diverse set of niches, from the rhizosphere to a human host. Some of the soil-dwelling pseudomonads, such as Pseudomonas protegens and Pseudomonas fluorescens, have been shown to inhibit specific bacterial and fungal phytopathogens (1, 2). In this study, we isolated and sequenced a potentially bioactive *Pseudomonas* sp. strain, DTU12.3, from leaf-covered soil in Denmark.

The DTU12.3 strain was initially isolated by diluting the collected soil sample, incubating it on selective *Pseudomonas* medium, and picking an individual fluorescent colony. 16S rRNA gene sequencing confirmed that the DTU12.3 strain belonged to the *Pseudomonas* genus. Prior to genomic DNA isolation, a liquid DTU12.3 culture was grown shaking overnight in lysogeny broth (LB) at 30°C. For Illumina sequencing, the Wizard genomic DNA purification kit (Promega) was used to isolate DNA, followed by generation of DNA libraries using a modified (half volume of each reagent) protocol of the Kapa HyperPlus library prep kit (Roche Molecular Systems) and sequencing on the Illumina MiSeq platform (300 cycles). For Nanopore sequencing, genomic DNA was isolated with the PureLink genomic DNA kit (Thermo Fisher Scientific), while DNA libraries were prepared with the rapid sequencing kit (Oxford Nanopore) and sequenced on the Nanopore MinION instrument (FLO-MIN106 flow cell). In total, 5,302,168 paired-end (2 × 150-bp) Illumina reads and 11,882 Nanopore reads (average read length, 10,881 bp; read length *N*_50_, 19,417 bp) were generated. Low-quality Illumina reads were trimmed with seqtk v1.2-r94, and Nanopore reads were trimmed with Porechop v0.2.2. Unicycler v0.4.1 was used for a hybrid assembly of the DTU12.3 genome using both Illumina and Nanopore reads (3). A single circular chromosome sequence was assembled, with a size of 6,268,469 bp, G+C content of 59.46%, and average read depth of 76-fold. The genome was annotated with the NCBI Prokaryotic Genome Annotation Pipeline and was predicted to have 5,795 genes, including 5,489 protein-coding genes, 19 rRNAs, 74 tRNAs, 4 noncoding RNAs, and 209 pseudogenes (4). Based on a method by Mulet et al. (5), the BLASTN comparison of the 16S rRNA gene, *rpoD*, *rpoB*, and *gyrB* sequences across *Pseudomonas* species revealed no matches above the species identity threshold (97%). To further support this finding, the online tool JSpeciesWS was used to calculate the average nucleotide identity (ANIb) of DTU12.3 against genomes of the 10 highest BLASTN comparison hits, and there were again no matches above the identity threshold (95%) (6). These analyses suggest that DTU12.3 could belong to a yet uncharacterized *Pseudomonas* species.

The bacterial production of secondary metabolites has been implicated in bioactivity against other bacterial and fungal species (1, 7). AntiSMASH v3.0 was used to identify eight putative secondary metabolite clusters in the genome of DTU12.3, namely, three nonribosomal peptide synthetase clusters, three bacteriocins, one arylpolyene, and one unnamed cluster (8). DTU12.3 exhibits growth inhibitory activity against the bacterial phytopathogens Xanthomonas campestrii and Dickeya solani *in vitro* (our unpublished data). The available genome sequence of *Pseudomonas* sp. strain DTU12.3 will potentially enable further discovery as well as functional characterization of specific genomic regions important for bioactivity against relevant plant pathogens.

### Data availability.

The complete genome sequence of *Pseudomonas* sp. strain DTU12.3 has been submitted to GenBank under the accession number CP027218. Raw sequencing reads have been deposited in the Sequence Read Archive (SRR6785587 and SRR6785588).
